# Chronic pain as a mediator in the falls-frailty association: Evidence from middle-aged and older ethnic minorities in Yunnan, China

**DOI:** 10.1016/j.tjfa.2025.100050

**Published:** 2025-05-16

**Authors:** Xuan Wen, Rui Deng, Xinping Wang, Chunyan Deng, Xiaoju Li, Yafang Zhang, Ying Chen, Yuan Huang

**Affiliations:** a School of Public Health, Kunming Medical University, Yunnan 650500, PR China; b Yunnan Provincial Key Laboratory of Public Health and Biosafety, Yunnan 650500, PR China; c Yunnan Provincial Key Laboratory of Cross-Border Infectious Disease Prevention and New Drug Development, Yunnan 650500, PR China

**Keywords:** Frailty, Chronic pain, Falls, Mediation effect, Ethnic minorities, Middle-aged and older adults

## Abstract

**Background:**

Falls are associated with an increased risk of frailty in middle-aged and older adults; however, the mediating role between falls and frailty remains underexplored, particularly among ethnic minority groups with distinct sociocultural and environmental exposures. Ethnic minority populations exhibit significant disparities in the prevalence of falls, frailty, and chronic pain compared to the majority. The primary objective of this study is to examine the relationship between falls and frailty in middle-aged and elderly individuals residing in Yunnan Province, China, with a specific emphasis on exploring the potential mediating effect of chronic pain across different ethnic groups.

**Methods:**

Employing a cross-sectional design, data were collected from July to December 2022 from adults aged ≥45 years in five ethnically diverse counties of Yunnan Province, China. Structured face-to-face interviews and stratified multistage cluster sampling were used for data collection. Baron and Kenny’s causal steps method was used to explore the mediating effect of chronic pain on the relationship between falls and frailty. Spearman correlation analysis, multiple linear regression models, and bootstrap method were used for data analysis.

**Results:**

A total of 2710 respondents participated in this study. The age distribution was as follows: 1161 (42.84 %) aged 45–59 years, 863 (31.85 %) aged 60–69 years, and 686 (25.31 %) aged 70 years or older. The sample comprised 1218 males (44.94 %) and 1492 females (55.06 %). The prevalence of falls among middle-aged and older individuals was 12.77 %, while the prevalence of frailty in the same population was observed to be 21.62 %. Spearman correlation analysis revealed significant positive association between chronic pain and both falls (*r* = 0.135, *P* < 0.05) as well as frailty (*r* = 0.383, *P* < 0.05). Frailty also exhibited a significant positive correlation with falls (*r* = 0.162, *P* < 0.05). After adjusting for all covariates, the total effect of falls on frailty was estimated to be 1.065 (95 % bootstrap *CI*: 0.804∼1.326), with a direct effect estimate of 0.797 (95 % bootstrap *CI*: 0.511∼1.083). The indirect effect of chronic pain on this association was found to be approximately one-quarter at 0.268 (95 % bootstrap *CI*: 0.170∼0.366). The subgroup analysis discovered differences in the mediating effects across different ethnic groups; specifically, the proportions mediated by chronic pain were found to be 28.2 %, 18.4 %, and 21.5 % for *Han* majority group, *Zhiguo* ethnic minorities, and other ethnic minority groups, respectively.

**Conclusion:**

This study provides valuable insights into the intricate association between frailty, falls, and chronic pain among middle-aged and older adults from diverse ethnic backgrounds in a western province of China. Effective management strategies targeting chronic pain and falls prevention could serve as crucial interventions to address frailty.

## 1 Introduction

Falls are a common unintentional event among older adults, often resulting in injuries that significantly impact health and quality of life. They have been widely recognized as a predominant challenge in geriatric nursing [1]. According to the Global Burden of Disease Study, the incidence, mortality, and disability-adjusted-life-years of falls in the Chinese population increased in rank from 27th in 1990 to 17th in 2017 [2]. Statistics show that in 2019, falls contributed significantly to the burden of disease and ranked as the second leading cause of injury-related death in China [3]. In addition to the immediate threat to life, falling also leads to various other health issues. Older adults who experience falls often face long-lasting physical and psychological consequences, with frailty being among the most common [4,5]. A cohort study indicating that seniors who experience falls are 1.92 times more likely to develop frailty [6]. Particularly noteworthy are the fall-induced consequences such as fractures, infections, and muscle injuries, which have independently been recognized as predictors of both the onset and progression of frailty [7]. This correspondence between falls and an increased likelihood of frailty in older adults has been further substantiated through comprehensive meta-analyses [8].

Chronic pain, presenting as a common outcome of falls [9], endures for extended duration, often spanning months or even years, thereby exerting a deleterious impact on an individual's quality of life and impeding their ability for movement [10], emotion management [11] and social contact [12]. In addition, chronic pain conditions such as knee osteoarthritis and low back pain can impair balance and mobility, significantly increasing the risk of recurrent falls [13]. This creates a vicious cycle wherein falls exacerbate pain, leading to further functional decline. Cohort studies have shown that obvious injuries and fractures sustained by older adults after a fall, or even soft tissue injuries, sprains, muscle strains, all can contribute to the development of chronic pain [14]. Moreover, community-dwelling elderly people who have experienced falls are particularly susceptible to the development of persistent chronic pain, with a considerable proportion of this group reporting severe discomfort [15]. Chronic pain makes older adults more vulnerable to an increased risk of depression and social isolation, which further leads to the exacerbation of frailty [16].

Extensive research has been conducted to investigate on the mediating function of chronic pain, with a primary focus on physical impairment [17], sleep quality [18], chronic diseases, and depression [19,20]. Concurrently, certain investigators have also explored the potential mediating influences of social participation [21], activity engagement [22] as mediating variables for falls and frailty. However, the relationship among falls, chronic pain, and frailty has not been extensively studied, it is still unclear whether chronic pain mediates the relationship between falls and frailty, especially among middle-aged and older adults in ethnic minority regions.

Yunnan Province accommodates 26 of China's 55 ethnic minorities [23], including 11 that are known as "*Zhiguo*" ethnic minorities, referring to the ethnic groups that have transitioned directly from primitive society to socialist society without undergoing democratic revolution. During the establishment of the People's Republic of China, these specific ethnic groups were still in an early stage of societal development. The Communist Party of China implemented specialized policies to facilitate their direct transition from primitive societies to socialism. As a result, eleven ethnic groups were officially recognized under this category: Wa, Pumi, Achang, Lisu, Lahu, Bulang, Jingpo, Nu, Jino De'ang and Drung people. These eleven ethnic minorities primarily reside in remote areas within Yunnan province in China where they have encountered significant poverty and resource shortages due to their geographical isolation [24]. Despite significant advancements during China's overall progress, these circumstances pose substantial challenges for improving the health of ethnic minorities. In recent years, as the aging population continues to grow, ensuring the health security of elderly individuals from different ethnic groups has become an integral part of China's pursuit towards achieving universal health coverage. This pressing need necessitates relevant research to investigate the factors influencing the health status of older people across diverse ethnicity. Due to cultural, occupational, and environmental factors, there are significant differences in fall rates among different ethnic groups [25]. Notably, in Yunnan Province, ethnic minorities exhibit shorter life expectancy compared to the Han majority and are therefore more likely to experience health issues such as falls and frailty during midlife [26]. Therefore, our specific focus lies on studying individuals aged 45 and above with the aim of gaining a deeper understanding of their distinct health needs and challenges.

The primary purpose of this study is to examine the correlation between falls and frailty in middle-aged and elderly people in Yunnan Province, China, with a particular focus on the potential mediating effect of chronic pain. To comprehensively understand the intricacies across various ethnicity, the study is designed with three main objectives: [1] to assess the association between chronic pain, falls and frailty among middle-aged and older adults in ethnic minority regions, [2] to investigate the mediating role of chronic pain on the relationship between falls and frailty, and [3] to explore potential disparities in the mediating effects among distinct ethnic groups, ultimately offering novel insights for elderly care strategies tailored to this vulnerable population.

## 2 Materials and methods

### 2.1 Study design

A cross-sectional design was used to collect data from July to December 2022 in Yunnan Province, China. The selection of study sites was based on geographical characteristics, economic development status, and the distribution of ethnic minority populations. Consequently, five counties representing diverse ethnic minority settlements and varying levels of economic development were chosen from the eastern, central and western regions of the province. Within each selected county, one township and two villages were randomly selected for respondents’ recruitment. The inclusion criteria for participants included individuals aged 45 years or older as of 2022 with a residency duration of at least three consecutive months in the local area and without significant intellectual or mental disorders. The recruitment of potential participants from each village was conducted using cluster sampling. All participants completed the survey under the guidance of interviewers. All participants were voluntary and had provided informed consent prior to face-to-face interviews using a structured questionnaire. Our interviewers, who possessed expertise in population-based surveys, underwent standardized training specifically tailored for this study. The sample size calculation was based on a fall prevalence rate of 12 % observed in the study population. Using a 95 % confidence level and a 5 % margin of error, the minimum required sample size for each ethnic group was calculated to be 163. Given that 12 ethnic groups were surveyed, the total minimum required sample size was determined to be 1956. A total of 2730 individuals were invited to participate in the survey. However, upon careful examination of the collected data, 15 participants were excluded due to missing key variables and 5 participants were excluded as their age was below 45 years. As a result, the final sample size for analysis comprised 2710 participants, including 814 *Han* majority (30.04 %), 1036 *Zhiguo* ethnic groups (38.23 %), and 860 other ethnic minorities (31.73 %).

### 2.2 Measurements

#### 2.2.1 Falls

In our study, participants were asked the following question, "Have you experienced any falls within the preceding 12 months?" The response options provided were binary (Yes or No) [27].

### 2.3 Frailty

The Tilburg Frailty Indicator (TFI), a standardized self-reported questionnaire with 15-item across three domains [28], was utilized to assess frailty. The physical domain consists of eight items encompassing aspects such as physical health, unexplained weight loss, difficulty in walking, balance, hearing problems, vision problems, and weak strength in hand, and physical tiredness. Each item is scored on a scale ranging from 0 to 8 points. The psychological domain includes four items assessing cognition, depressive symptoms, anxiety levels and coping mechanisms. These items are also scored on a scale ranging from 0 to 4 points. Lastly, the social domain includes three items evaluating living arrangements, social relationships and social support with scores ranging from 0 to 3 points. The total TFI score ranges from 0 to 15. An individual with a score of ≥5 is considered frailty: higher scores indicate greater degrees of frailty. Previous studies have demonstrated the reliability and validity of the TFI among the Chinese population [29]. In this study, to assess the internal consistency of the frailty scale, we calculated the Cronbach's alpha coefficient based on the covariance among all items of the scale. The resulting Cronbach's alpha coefficient for the TFI was 0.735.

### 2.4 Chronic pain

In our survey, respondents were asked to indicate whether they frequently experienced discomfort due to pain over the past three months with binary options (Yes or No). To further evaluate the intensity of participants’ pain, we utilized a numerical rating scale (NRS) consisting of 10 points. Participants were instructed to select a score ranging from 0 to 10 to describe the severity of their pain [30]. The assigned values for pain points were interpreted as follows: 0 denoted no pain, while scores between 1 and 3 represented mild pain, scores between 4 and 6 indicated moderate pain, and scores between 7 and 10 signified severe pain [31].

### 2.5 Covariates

The covariates consist of two aspects: demographic characteristics and health-related factors. Demographic characteristics encompass ethnicity, age (45–59, 60–69, and ≥70), sex, marital status (whether there is a partner), education level (illiterate, uncompleted primary school, completed primary school, middle school or above), occupation (farmers, non-farm workers), and monthly household income (<1000 RMB, 1000–2999 RMB, 3000–4999 RMB, ≥5000 RMB). To assess the disparities among ethnic minority groups, we categorized them into three groups: *Han* majority population, *Zhiguo* ethnic minorities, and other ethnic minorities. Among the 11 officially recognized *Zhiguo* ethnic minorities, we have selected six as representatives for this study: *Jino, Nu, Bulang, Lisu, Lahu* and *Wa*. Other ethnic minorities included in this study consist of *Dai, Bai, Zhuang, Yi*, and *Hani*.

Covariates regarding health-related factors at the individual level included chronic diseases, smoking behavior, drinking habit, and activities of daily living (ADL) as they have been previously associated with frailty in older adults [32]. Respondents were asked to report if they had any of the following chronic conditions: diabetes mellitus, hypertension, dyslipidemia, heart disease, cerebrovascular disease, chronic gastritis, chronic lung disease, chronic nephritis, rheumatoid arthritis, intervertebral disc disease, osteoporosis, ventilation disorder, hyperthyroidism, hypothyroidism, cancer, leukemia, chronic anemia, or other chronic diseases. Chronic diseases were defined as having at least one chronic disease (Yes or No). Smoking status was determined by whether respondents currently smoked or had ever smoked (Yes or No). Alcohol consumption within the past week was also assessed (Yes or No). The Activity of Daily Living Scale [33] was used to evaluate the ADL status. The total score ranges from 14 to 56 with higher scores indicating greater impairment in daily living functionality for the participant. A score of 14 indicates absence of disabilities while a range of 15–21 represents mild disabilities and a score above or equal to 22 signifies severe disabilities.

### 2.6 Statistical analyses

The demographic characteristics and health-related factors of the respondents were described using frequencies (percentages). C*hi-square* tests were used to compare differences between groups. Spearman correlation analysis was conducted to determine the correlation between chronic pain, falls and frailty. Baron and Kenny’s causal steps method was used to explore the mediating effect of chronic pain on the relationship between falls and frailty [34]. Following the proposed procedures of this method, a three-step estimation approach was applied to analyze the mediating variable. The mediation effect analysis needs to meet the following conditions: [1] falls was significantly associated with frailty (Total effect; Path c); [2] falls was significantly associated with chronic pain (Path a); [3] controlling for falls, chronic pain was significantly associated with frailty (Path b); [4] the relationship between falls and frailty was reduced (Direct effect, Path c’) when controlling for chronic pain (Indirect effect, *a* × *b*). Full mediation occurs when inclusion of the mediation variable reduces the observed relationship between independent variable and dependent variable to zero. Partial mediation occurs when inclusion of mediation effect weakens the observed relationship between independent and dependent variable. Recent studies have shown that bootstrap confidence interval is one of the most reliable tests for conducting a mediation analysis [35]. We set bootstrap confidence interval (*CI*) at 95 % with 5000 bootstrap samples. If zero is not included in 95 % *CI*, it indicates a significant mediating effect [36]. In our study, we chose to employ a linear regression model due to the inherent characteristics of the dependent variable. Linear regression models were used for conducting multivariable regression analyses involving continuous outcomes such as chronic pain score and frailty score. Compared to logistic regression, linear regression avoids probability transformation and enables a more direct modeling of the linear relationship between the dependent and independent variables, potentially yielding more precise results [37]. All analyses were performed using Stata v.15 software with statistical significance considered at *p*-value < 0.05.

## 3 Results

### 3.1 Demographic and health characteristics among middle-aged and elderly people

A total of 2710 respondents participated in this study. The age distribution was as follows: 1161 (42.84 %) aged 45–59 years, 863 (31.85 %) aged 60–69 years, and 686 (25.31 %) aged 70 years or older. The sample comprised 1218 males (44.94 %) and 1492 females (55.06 %). The prevalence of falls among middle-aged and older individuals was 12.77 %, while the prevalence of frailty in the same population was observed to be 21.62 %. According to Table 1, it is evident that mild pain was experienced by 30.55 % of respondents, moderate pain was reported by 24.91 % a smaller proportion (1.88 %) reported severe pain. The three most frequently reported pain locations were the waist (37.45 %), knees (28.76 %), and neck (22.01 %). For fall incidence, no statistically significant difference was observed between the elderly (13.7 %) and middle-aged (11.5 %) groups (*P* > 0.05). For Chronic pain prevalence, a statistically significant difference existed between the elderly (63.5 %) and middle-aged individuals (49.2 %) (*P* < 0.05). Regarding frailty prevalence, a statistically significant difference was noted between the elderly (26.9 %) and middle-aged (14.6 %) groups (*P* < 0.05). Notably, statistically significant differences (*P* < 0.05) were observed among ethnic groups with respect to age, education, occupation, monthly household income, prevalence of chronic diseases, drinking habits, and ADL. Furthermore, there were also ethnic-based disparities (*P* < 0.05) noted in terms of the incidence of falls as well as the prevalence of chronic pain and frailty.

**Table 1:** Demographic characteristics and health status of middle-aged and elderly adults by ethnic groups [n (%)].

Variable	Total (*n* = 2710)	Ethnic groups			*χ^2^*	*P*
		*Han* majority (*n* = 814)	*Zhiguo* minorities (*n* = 1036)	Other minorities (*n* = 860)		
**Demographics**						
Age						
45–59	1161(42.84)	298(36.61)	513(49.52)	350(40.70)	87.385	<0.001
60–69	863(31.85)	224(27.52)	342(33.01)	297(34.53)		
≥70	686(25.31)	292(35.87)	181(17.47)	213(24.77)		
Sex						
Male	1218(44.94)	353(43.37)	482(46.53)	383(44.53)	1.924	0.382
Female	1492(55.06)	461(56.63)	554(53.47)	477(55.47)		
Marital status						
Married	2054(75.79)	630(77.40)	769(74.23)	655(76.16)	2.587	0.274
Single	656(24.21)	184(22.60)	267(25.77)	205(23.84)		
Education level						
Illiterate (Never received an education)	1098(40.52)	253(31.08)	459(44.30)	386(44.88)	71.904	<0.001
Uncompleted primary school	586(21.62)	176(21.62)	197(19.02)	213(24.77)		
Completed primary school	468(17.27)	159(19.54)	174(16.80)	135(15.70)		
Middle school or above	558(20.59)	226(27.76)	206(19.88)	126(14.65)		
Occupation						
Farmers	1902(70.18)	398(48.89)	833(80.41)	671(78.02)	253.291	<0.001
Non-farm workers	808(29.82)	416(51.11)	203(19.59)	189(21.98)		
Monthly household income (Chinese yuan/RMB)						
<1000	623(22.99)	165(20.27)	313(30.21)	145(16.86)	80.704	<0.001
1000–2999	848(31.29)	231(28.38)	338(32.63)	279(32.44)		
3000–4999	722(26.64)	235(28.87)	207(19.98)	280(32.56)		
≥5000	517(19.08)	183(22.48)	178(17.18)	156(18.14)		
**Health-related factors**						
Chronic diseases						
Yes	1572(58.01)	538(66.09)	594(57.34)	440(51.16)	38.581	<0.001
No	1138(41.99)	276(33.91)	442(42.66)	420(48.84)		
Smoking						
Yes	906(33.43)	262(32.19)	351(33.88)	293(34.07)	0.818	0.664
No	1804(66.57)	552(67.81)	685(66.12)	567(65.93)		
Drinking						
Yes	711(26.24)	160(19.66)	313(30.21)	238(27.67)	27.595	<0.001
No	1999(73.76)	654(80.34)	723(69.79)	622(72.33)		
ADL						
No disability (Score 14)	1611(59.45)	525(64.50)	579(55.89)	507(58.95)	15.963	<0.001
Mild disability (Score 15–21)	976(36.01)	253(31.08)	403(38.90)	320(37.21)		
Severe disability (Score ≥22)	123(4.54)	36(4.42)	54(5.21)	33(3.84)		
Falls						
Yes	346(12.77)	127(15.60)	132(12.74)	87(10.12)	11.300	0.004
No	2364(87.23)	687(84.40)	904(87.26)	773(89.88)		
Chronic pain						
No pain (Score 0)	1156(42.66)	336(41.28)	392(37.84)	428(49.77)	46.988	<0.001
Mild pain (Score 1–3)	828(30.55)	239(29.36)	344(33.20)	245(28.49)		
Moderate pain (Score 4–6)	675(24.91)	222(27.27)	268(25.87)	185(21.51)		
Severe pain (Score 7–10)	51(1.88)	17(2.09)	32(3.09)	2(0.23)		
Frailty						
Yes	586(21.62)	191(23.46)	272(26.25)	123(14.30)	41.938	<0.001
No	2124(78.38)	623(76.54)	764(73.75)	737(85.70)		

### 3.2 Correlation of chronic pain, falls and frailty

The Spearman correlations (*r*) between three variables, namely chronic pain, falls, and frailty are presented in Table 2. For the entire population, a statistically significant positive correlation was observed between chronic pain and falls (*r* = 0.135, *p* < 0.05). Similarly, a significant positive correlation was found between chronic pain and frailty (*r* = 0.383, *p* < 0.05). Additionally, there was a significant positive correlation of 0.162 (*p* < 0.05) between falls and frailty as well. In different ethnic groups, significant positive correlations were also observed among chronic pain, falls, and frailty.

**Table 2:** Correlations between chronic pain, falls and frailty (*r*).

Variables	Whole sample	*Han* majority	*Zhiguo* minorities	Other minorities
Chronic pain - Falls	0.135*	0.185*	0.088*	0.133*
Chronic pain - Frailty	0.383*	0.369*	0.403*	0.339*
Falls - Frailty	0.162*	0.197*	0.138*	0.142*

Note: * *P* < 0.05.

### 3.3 Mediating effect of chronic pain on the relationship between falls and frailty

**Fig. 1:**
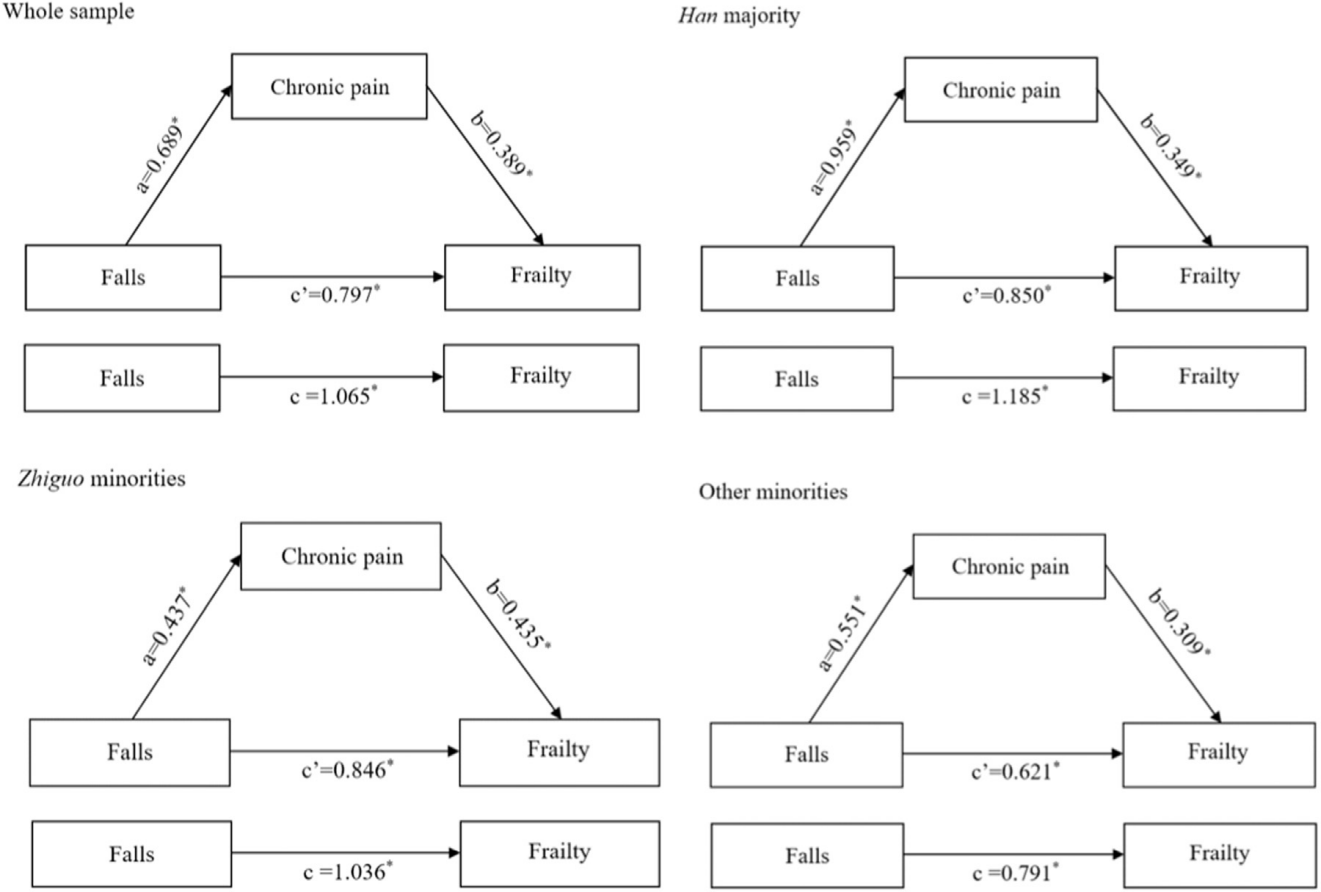
Model of the mediating effect of chronic pain on the relationship between falls and frailty. Note: *P < 0.05.

The regression model results based on Baron and Kenny's causal steps method were presented in Table 3 and Fig. 1. After adjusting for all the covariates, the association between falls and chronic pain as found to be significant (*β*=0.689, 95 %*CI*: 0.458∼0.919) for path a. Similarly, controlling for falls, chronic pain showed a significant association with frailty (*β=*0.389, 95 %*CI*: 0.349∼0.429) for path b. Additionally, falls exhibited a significant association with frailty (*β=*1.065, 95 %*CI*: 0.804∼1.326) for path c. After introducing chronic pain into the model as a mediator (path c’), the *β* coefficient of falls on frailty decreased (*β*=0.797, 95 % *CI*: 0.551∼1.043), suggesting a potential mediating effect of chronic pain on the relationship between falls and frailty. Furthermore, this mediating effect was confirmed across different ethnic groups.

**Table 3:** Analysis of mediating effect of chronic pain.

		Pathway	Whole sample (*N* = 2710)	*Han* majority (*N* = 814)	*Zhiguo* minorities (*N* = 1036)	Other minorities (*N* = 860)
			*β* (95 % *CI*)	*β* (95 % *CI*)	*β* (95 % *CI*)	*β* (95 % *CI*)
Fall	Frailty	c	1.065*(0.804∼1.326)	1.185*(0.737∼1.633)	1.036*(0.595∼1.477)	0.791*(0.336∼1.246)
Fall	Chronic pain	a	0.689*(0.458∼0.919)	0.959*(0.563∼1.355)	0.437*(0.043∼0.831)	0.551*(0.147∼0.955)
Fall	Frailty	c’	0.797*(0.551∼1.043)	0.850*(0.418∼1.282)	0.846*(0.438∼1.253)	0.621*(0.181∼1.061)
Chronic pain		b	0.389*(0.349∼0.429)	0.349*(0.274∼0.424)	0.435*(0.372∼0.499)	0.309*(0.236∼0.382)

Note: *β* Adjusted for age, sex, marriage, education level, occupation, monthly household income, chronic diseases, smoking, drinking, ADL. * *P* < 0.05.

### 3.4 Bootstrap test results

We used bootstrap testing to further validate the mediating role of chronic pain in the relationship between falls and frailty. For the entire population, the total effect of falls on frailty was 1.065 (95 % bootstrap *CI*: 0.804∼1.326), with a direct effect of 0.797 (95 % bootstrap *CI*: 0.511∼1.083) and an indirect effect of 0.268 (95 % bootstrap *CI*: 0.170∼0.366). Chronic pain partially mediated the effect of falls on frailty, explaining that approximately 25.2 % of this effect can be attributed to mediating effects. In addition, we conducted separate analyses to examine the mediating effect of chronic pain on the relationship between falls and frailty across different ethnic groups, aiming to evaluate potential differences in this mediation among these groups. The results showed that within the *Han* population, chronic pain accounted for approximately 28.2 % (indirect effect=0.335; 95 % bootstrap *CI*: 0.171∼0.499) of the total effect as a mediator between falls and frailty. In *Zhiguo* ethnic populations, chronic pain contributed to about 18.4 % (indirect effect=0.190; 95 % bootstrap *CI*: 0.008∼0.372), of the total effect as mediator between falls and frailty. Among other minority populations, chronic pain explained approximately 21.5 % (indirect effect=0.170; 95 % bootstrap *CI*: 0.025∼0.315) of the total effect as a mediator between falls and frailty. Detailed information is presented in Table 4. In the middle-aged group (45–59 years), chronic pain mediated 21.4 % of the total effect (indirect effect=0.246, 95 % bootstrap *CI*: 0.090∼0.401). In the elderly group (≥60 years), chronic pain exhibited a stronger mediating effect, accounting for 27.6 % of the total effect (indirect effect=0.271, 95 % bootstrap *CI*: 0.144∼0.397). Detailed data is provided in the appendix.

**Table 4:** Bootstrap test results for different populations.

Populations	Effect		*SE*	95 % bootstrap *CI*	Mediation effect (%)
Whole sample (*N* = 2710)	Total effect	1.065	0.133	0.804∼1.326	-
	Direct effect	0.797	0.146	0.511∼1.083	-
	Indirect effect	0.268	0.050	0.170∼0.366	25.2 %
**Stratified by ethnic group**					
*Han* majority (*N* = 814)	Total effect	1.185	0.228	0.737∼1.633	-
	Direct effect	0.850	0.248	0.364∼1.337	-
	Indirect effect	0.335	0.084	0.171∼0.499	28.2 %
*Zhiguo* minorities (*N* = 1036)	Total effect	1.036	0.225	0.595∼1.477	-
	Direct effect	0.846	0.231	0.393∼1.299	-
	Indirect effect	0.190	0.093	0.008∼0.372	18.4 %
Other minorities (*N* = 860)	Total effect	0.791	0.232	0.336∼1.246	-
	Direct effect	0.621	0.258	0.116∼1.126	-
	Indirect effect	0.170	0.074	0.025∼0.315	21.5 %

Note: Effect, standardized regression coefficient. *SE*, standard error.

## 4 Discussion

The findings revealed a high prevalence of falls, chronic pain, and frailty within this population, with a significant and noteworthy correlation observed between chronic pain, falls, and frailty. Specifically, it was found that chronic pain partially mediated the relationship between falls and frailty, exhibiting variations across diverse ethnic groups.

A striking revelation pertains to the higher prevalence of frailty symptoms (21.62 %) among middle-aged and senior participants compared to a previous study conducted in the southwestern region, which reported a frailty prevalence of 12.7 % among elderly individuals living in community settings [38]. Despite the disparate age ranges, this study highlights a considerably high occurrence of frailty among individuals aged 45 and above residing in ethnic minority areas. This discernment engenders a compelling proposition that frailty may manifest itself at an earlier chronological phase than hitherto believed and extend beyond the customary threshold of those aged 60 and above [39]. However, it should be noted that the higher prevalence observed in this study may be ascribed to a pronounced proportion of ethnic minorities inhabiting in rural areas, particularly among the *Zhiguo* ethnic minorities. This is where both the proportion of middle-aged inhabitants and the prevalence of frailty attain zenith. The national census data indicates that *Zhiguo* ethnic minorities experienced shorter life expectancy than *Han* majority population, implying an increased likelihood of experiencing frailty at younger ages within these ethnic groups [40]. In light of these findings, there exists a compelling rationale for further exploration into determinants influencing the onset, progression and deterioration of frailty among middle-aged and older adults from less developed areas. Tailored interventions should be provided to specific minority groups in order to address their unique needs and aging trajectory. Effective intervention measures, such as physical exercise, nutritional intake, and social support, can be implemented to cater to the distinct challenges faced by these minority groups [41].

Consistent with previous findings [42,43], the present results suggest a significant association between falls and frailty among middle-aged and older adults irrespective of their ethnicity. In addition to physical injuries such as fractures and sprains resulting from falls, prolonged bed rest after a fall could negatively impact the daily activities and social interactions of fallen person, thereby further exacerbating the occurrence of frailty [44]. Hence, the imperative of fall prevention assumes paramount significance within the broader framework of frailty prevention. However, it is worth noting that participants in this study inhabited in rural areas and exhibited a low level of education. The determinants engendering their susceptibility to falls may differ from their urban or community-dwelling counterparts. Engaging in strenuous physical labor can lead to comparatively elevated levels of muscle quality and strength [45]. Nevertheless, as these individuals advance in age, they are susceptible to experiencing fatigue and muscle exhaustion due to the prolonged nature of their physical labor. This can be concomitant with the onset of pain in various regions of the body, thereby elevating their vulnerability to falls [46].

As suggested in previous study, the perception of falling among elderly individuals can substantively influence the incidence of falls [47]. Rural-dwelling elderly individuals tend to perceive falls as an inherent aspect of aging rather than a latent health peril. This perspective could engender a reluctance amongst older adults to seek assistance or adopt proactive preventive measures following a fall, consequently increasing their vulnerability to frailty [48].

Moreover, the accelerated process of urbanization over the preceding three decades in China has witnessed a substantial influx of large-scale migrant laborers into metropolises, coupled with a continuous upsurge in solitary living among elderly individuals nestled in rural areas or small towns [49]. This population often encounters heightened inconveniences, difficulties, and health risks compared to those residing with their adult children [50]. Consequently, they may exhibit an elevated proactivity for medical services, such as fall prevention and post-fall care, the facets that are currently insufficiently addressed by the prevailing healthcare system.

Our study unveils a crucial facet of the relationship between falls and frailty — the mediating role of chronic pain. Specifically, we observed that chronic pain mediates 25.2 % of the overall effect of falls on frailty, underscoring the significance of chronic pain in influencing frailty development. Previous research has shown that middle-aged and older adults who have experienced falls are more prone to developing chronic pain as a result of physical injury [32]. Furthermore, Chronic pain serves as a predictive indicator for frailty in middle-aged and elderly individuals, with higher pain intensity, chronic widespread pain, and greater pain interference associated with increased vulnerability to frailty [51].

Importantly, after accounting for the presence of chronic pain, the association between falls and frailty decreased from 1.065 (total effect) to 0.767 (direct effect), signifying a potential avenue for intervention. Integrating strategies for managing chronic pain into fall prevention approaches targeted at middle-aged and elderly individuals could play a pivotal role in mitigating frailty onset.

Subgroup analysis revealed variation in mediating effects among different ethnic groups. These disparities can be attributed to several factors supported by existing literature. Firstly, differences in gene, physiology, and metabolism across racial and ethnic groups can influence pain sensitivity, tolerance, and reporting severity [52]. Social context and lifestyle also play significant roles. For instance, due to geographic and economic constraints, the dietary habits of ethnic minority populations often lack essential nutrients such as Vitamin D and calcium [53], leading to weakened bones and muscles and consequently increasing the risk of falls and frailty [54]. Additionally, diverse exercise habits and medication use within different ethnic groups can differentially impact disease risks and chronic pain recovery [55]. Some ethnic minorities typically rely on agriculture for their livelihoods. Agricultural labor, which involves activities such as tilling the land, planting crops, and harvesting, requires continuous physical movement. This not only enhances muscle strength but also significantly improves balance [56]. Moreover, strong family and community support in ethnic minorities can facilitate timely care and recovery following falls or pain episodes. However, these benefits are often hindered by systemic barriers such as limited healthcare access and geographic isolation, which may delay or compromise the quality of care received [57]. Notably, our study indicated a higher tendency for alcohol consumption among the *Zhiguo* ethnic minorities, which could potentially exert an analgesic effect on chronic pain, alleviating pain sensations [58].

Regarding different age groups, the greater proportion of the mediated effect observed in the elderly group strongly suggests that as individuals progress into the senior phase of life, chronic pain becomes into a more crucial determinant in the association between falls and the frailty onset. One plausible explanation for this disparity is closely linked to the profound physiological changes inherently associated with aging [59]. However, in the middle-aged group, the direct mediating effect of chronic pain between falls and frailty remains at 21.4 %, indicating that chronic pain management in middle-aged individuals can not be overlooked.

While further research is essential to comprehensively elucidate the diverse impacts of chronic pain among different ethnic groups, our preliminary findings suggest that addressing chronic pain may hold varying degrees of importance in reducing frailty risk across these groups. This underscores the need for tailored interventions encompassing dietary therapy, medication management, physical therapy, and appropriate exercise regimens to effectively decelerate frailty progression.

### 4.1 Limitations

Our study has certain limitations that should be acknowledged. Firstly, our study used a face-to-face household survey method. Despite our best efforts to ensure the accuracy of the questionnaires, it is impossible to completely eliminate the potential for recall bias. Respondents were required to provide information on aspects such as chronic illnesses and household income, which may be susceptible to subjective memory and interpretation biases. In addition, the assessment of pain and frailty relies on structured self-assessment questionnaires, which may potentially impact the precision of results. To ensure familiarity and maximize measurement data reliability, we have implemented well-recognized scales and provided investigators with rigorous training. Secondly, although we collected data on pain severity and location, our mediation analysis focused exclusively on pain severity, potentially overlooking the specific impact of pain location on falls and frailty. Additionally, the absence of data on fall frequency limits our understanding of their cumulative impact on frailty progression. Future studies should incorporate both pain location and fall frequency to provide a more comprehensive understanding of these relationships. Thirdly, our study predominantly targeted an elderly population, potentially introducing language and communication barriers. To mitigate this issue, we enlisted assistance from family members of respondents for translation and communication purposes in order to optimize questionnaire quality. However, despite these measures being taken into account, we cannot entirely dismiss the potential impact of communication issues on data validity. Lastly, our study utilized cross-sectional data which limits establishing causal relationships between variables. Future longitudinal studies can further explore causal associations between these variables while considering additional potential confounding factors and temporal aspects.

## 5 Conclusion

The present study, conducted in a western province of China, involved a cohort of 2710 middle-aged and older adults, representing a diversity of ethnic backgrounds. A noteworthy finding emerges from this study, revealing that approximately one-fifth of the participants exhibited frailty, while roughly one-tenth experienced falls within the preceding 12 months. These findings highlight the interconnections between falls, chronic pain, and frailty among this specific population. Of particular significance, chronic pain emerged as a partial mediator in the relationship between falls and frailty with statistically significant effects observed across three subgroups by ethnic identity. In summary, this study provides valuable insights into the intricate nexus between frailty, falls, and chronic pain among middle-aged and older adults from diverse ethnic backgrounds in a western province of China. Consequently, this study underscores the potential utility of deploying effective management strategies targeting chronic pain and falls prevention as pivotal interventions to address frailty within this demographic.

## Funding

This study was supported by the Yunnan Fundamental Research Projects (202401AT070178), Philosophy and Social Science Innovation Team of Yunnan Province (2024CX08), Provincial Talent Program for Young Scholar and Technical Reserve Personnel (202305AC160046) and First-Class Discipline Team of Kunming Medical University (2024XKTDTS16).

## Declarations

Ethics approval and consent to participate

This study was approved by the ethics committee of the 10.13039/501100003996Kunming Medical University (NO. KMMU2021MEC095). At the beginning of the research, the researchers explained the project to the participants. The information included the aims of the study, potential advantages and disadvantages of participation, the expected benefits of carrying out the research, principles of privacy and confidentiality, and a declaration of voluntary participation. Participants were also informed that they could withdraw from the study at any time. The study was conducted in accordance with the Declaration of Helsinki, and all participants signed informed consent forms.

## Availability of data and materials

The datasets used and/or analyzed during the current study available from the corresponding author on reasonable request.

## Consent for publication

Not applicable.

## CRediT authorship contribution statement

**Xuan Wen:** Writing – original draft, Investigation, Formal analysis, Data curation, Conceptualization. **Rui Deng:** Writing – review & editing, Writing – original draft, Investigation. **Xinping Wang:** Investigation, Data curation. **Chunyan Deng:** Writing – original draft, Investigation. **Xiaoju Li:** Investigation, Data curation. **Yafang Zhang:** Investigation, Data curation. **Ying Chen:** Writing – review & editing, Supervision, Methodology. **Yuan Huang:** Writing – review & editing, Supervision, Methodology.

## Declaration of competing interest

The authors declare that they have no known competing financial interests or personal relationships that could have appeared to influence the work reported in this paper.

## Appendix Supplementary materials

Image, application 1
