# Supplementary material for: Chronic pain as a mediator in the falls-frailty association: Evidence from middle-aged and older ethnic minorities in Yunnan, China
Source: J Frailty Aging. 2025 May 16;14(4):100050. doi: 10.1016/j.tjfa.2025.100050 (PMC12184009; doi:10.1016/j.tjfa.2025.100050)
Supplement: Supplementary file 1 [file mmc1.docx]

**Appendix:**

**Appendix table 1** Analysis of mediating effect of chronic pain

|  | | Pathway | Whole sample  (N=2710) | Middle-aged group  (N=1161) | Elderly group  (N=1549) |
| --- | --- | --- | --- | --- | --- |
|  |  |  | *β* (95% *CI*) | *β* (95% *CI*) | *β* (95% *CI*) |
| Fall | Frailty | c | 1.065^*^(0.804~1.326) | 1.147^*^(0.774~1.520) | 0.380^*^(0.622~1.337) |
| Fall | Chronic pain | a | 0.689^*^(0.458~0.919) | 0.644^*^(0.292~0.995) | 0.697^*^(0.390~1.003) |
| Fall | Frailty | c’ | 0.797^*^(0.551~1.043) | 0.901^*^(0.551~1.251) | 0.709^*^(0.370~1.049) |
| Chronic pain |  | b | 0.389^*^(0.349~0.429) | 0.328^*^(0.325~0.439) | 0.388^*^(0.333~0.443) |

Note: *β* Adjusted for ethnic group, sex, marriage, education level, occupation, monthly household income, chronic diseases, smoking, drinking, ADL. ^*^ *P*<0.05.

**Appendix table 2.** Bootstrap test results for different populations

| Populations | Effect | | *SE* | 95% bootstrap *CI* | Mediation effect (%) |
| --- | --- | --- | --- | --- | --- |
| Whole sample (N=2710) | Total effect | 1.065 | 0.133 | 0.804~1.326 | - |
|  | Direct effect | 0.797 | 0.146 | 0.511~1.083 | - |
|  | Indirect effect | 0.268 | 0.050 | 0.170~0.366 | 25.2% |
| **Stratified by age group** |  |  |  |  |  |
| Middle-aged group (N=1161) | Total effect | 1.147 | 0.190 | 0.774~1.520 | - |
|  | Direct effect | 0.901 | 0.212 | 0.484~1.317 | - |
|  | Indirect effect | 0.246 | 0.079 | 0.090~0.401 | 21.4% |
| Elderly group (N=1549) | Total effect | 0.980 | 0.182 | 0.622~1.337 | - |
|  | Direct effect | 0.709 | 0.194 | 0.329~1.049 | - |
|  | Indirect effect | 0.271 | 0.065 | 0.144~0.397 | 27.6% |

Note: Effect, standardized regression coefficient. *SE*, standard error.


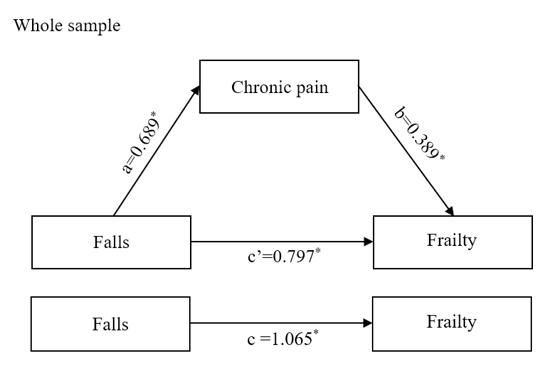

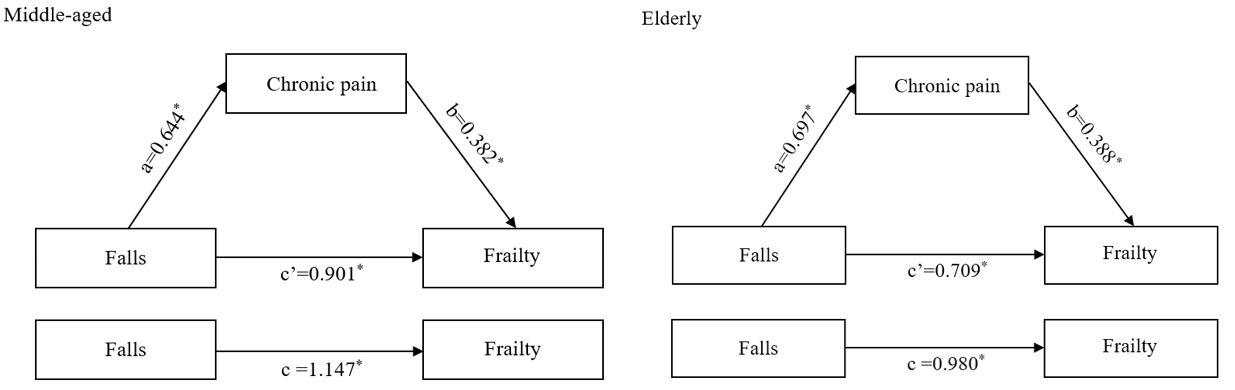


**Appendix figure 1** Model of the mediating effect of chronic pain on the relationship between falls and frailty.

Note: ^*^*P*<0.05.
